# Palinacusia: Regarding a peculiar clinical case

**DOI:** 10.1192/j.eurpsy.2025.1864

**Published:** 2025-08-26

**Authors:** R. Ramallo Castillo, M. Pérez Sosa, M. Vázquez Delgado, C. Sánchez Martínez, J. M. Duro Garrido

**Affiliations:** 1 Clinical Management Unit of Mental Health, Virgen Macarena University Hospital, Seville, Spain

## Abstract

**Introduction:**

A 44-year-old woman with a history of migraine, ulcerative colitis, obesity, and hypertension. She experienced a depressive episode that resolved completely in 2013. Hospitalized in 2020 and 2021 for psychotic symptoms (auditory pseudo-hallucinations) resistant to treatment, requiring bilateral ECT (LOW05, dose titration up to 45% - 227 mC-) and initiation of clozapine. In October 2021, she was enrolled in a monthly ECT maintenance program, maintaining a period of euthymia without psychotic symptoms until September 2022. At that point, she began to present anxiety secondary to auditory perseveration: after an auditory stimulus, the patient verbalizes an echo of that sound outside her head, which lasts for hours or even days.

**Objectives:**

Perform a differential diagnosis between schizoaffective disorder (given the persistence of psychotic symptoms despite euthymia) and palinacusia. Additionally, monitor the patient’s progress and address the described psychopathology according to current scientific evidence.

**Methods:**

Systematic review of the existing literature on the etiology, pathophysiology, and therapeutic approach to palinacusia. Clinical follow-up of the patient’s progression.

**Results:**

Various complementary tests are performed: normal blood tests (complete blood count, biochemistry, serologies, autoimmune markers, thyroid function). Clozapine levels: 315 ng/ml; norclozapine 189 ng/ml; ratio 1.6 (all within normal range). EEG: overload of slow activity over bilateral temporal regions. No postictal activity. MRI: slight cortico-subtemporal atrophic changes with a temporal predominance. No mesial sclerosis foci observed.

Since October 2023, different antiepileptic drugs have been trialed (topiramate, eslicarbazepine, and valproic acid). Additionally, the patient continues in a monthly maintenance ECT program (bilateral, LOW05, reaching a dose of 90%). As a result, the patient shows slow progress, with no remission of the described symptoms, although there is a temporary reduction in intensity (February 2024).

**Conclusions:**

The term “palinacusia” was described by Jacobs in 1971 as auditory perseveration (following a trigger and with neutral content). It is associated with various etiologies, primarily convulsive phenomena, and it remains debated whether it is ictal or postictal. The temporal lobe’s GTS is typically affected. To date, it has been described in 43 patients, and only in two patients with psychosis. The case described began after treatment with ECT, which raises the possibility of its pathophysiological involvement (difficulty in postictal suppression capacity). Currently, after discontinuing ECT treatment in March 2024 due to the possibility that it was exacerbating the palinacusia, and after trialing the aforementioned antiepileptic drugs, no remission of symptoms has been achieved.

**Disclosure of Interest:**

R. Ramallo Castillo Employee of: Internal medicine resident in the Andalusian Health System, M. Pérez Sosa Employee of: Specialist doctor in the Andalusian Health System, M. Vázquez Delgado Employee of: Specialist doctor in the Andalusian Health System, C. Sánchez Martínez Employee of: Internal medicine resident in the Andalusian Health System, J. M. Duro Garrido Employee of: Internal medicine resident in the Andalusian Health System

